# Latin America: Situation and preparedness facing the multi-country human monkeypox outbreak

**DOI:** 10.1016/j.lana.2022.100318

**Published:** 2022-07-06

**Authors:** Alfonso J. Rodriguez-Morales, Gustavo Lopardo, Sergio Verbanaz, Tomas Orduna, Susana Lloveras, Jose María Azeñas-Burgoa, Juan Pablo Escalera-Antezana, Lucia Elena Alvarado-Arnez, Alexandre Naime Barbosa, Fredi Diaz-Quijano, Sergio Cimerman, Tânia do Socorro Souza Chaves, Andrea G. Rodriguez-Morales, Cecilia Perret, Claudio A. Méndez, Jorge A. Riera, D. Katterine Bonilla-Aldana, German Camacho-Moreno, Henry Mendoza, Ivan Arturo Rodriguez-Sabogal, Jose Oñate, Angel A. Escobedo, Monica Thormann, Yori Roque, Patricia Gabriela Zambrano, Yenddy Carrero, Nancy Sandoval, Lysien Zambrano, Carlos Franco-Paredes, Enrique Chacon-Cruz, Iván Lopez-Delgado, Cesar Cuadra-Sánchez, Monica Pachar-Flores, Ricardo Correa, Hernan D. Rodriguez-Enciso, Veronica Rotela-Fisch, Julio Maquera-Afaray, Percy Herrera-Añazco, Vicente Benites-Zapata, Eduardo Savio-Larriera, Juan David Ramírez, Alberto Paniz-Mondolfi, Alejandro Risquez, David A. Forero-Peña, Jaime R. Torres, Jose Antonio Suarez

**Affiliations:** a Grupo de Investigación Biomedicina, Faculty of Medicine, Fundación Universitaria Autónoma de las Américas, Pereira, Risaralda, Colombia; b Master of Clinical Epidemiology and Biostatistics, Universidad Científica del Sur, Lima, Perú; c Latin American network of MOnkeypox VIrus research (LAMOVI), Pereira, Risaralda, Colombia; d Cátedra de Enfermedades Infecciosas, University of Buenos Aires, Buenos Aires, Argentina; e Servicio de Infectología, Hospital Británico de Buenos Aires. Buenos Aires, Argentina; f Hospital de Enfermedades Infecciosas F. J. Muñiz, Buenos Aires, Argentina; g Hospital Clínico Viedma, Cochabamba, Bolivia; h Direction, Centros de Salud de Primer Nivel, Secretaría de Salud, Gobierno Autónomo Municipal de Cochabamba, Cochabamba, Bolivia; i National Research Coordination, Franz Tamayo University (UNIFRANZ), La Paz, Bolivia; j Infectious Diseases Department, Botucatu Medical School, UNESP; Brazilian Society for Infectious Diseases, São Paulo, SP, Brazil; k Departamento de Epidemiologia, Faculdade de Saúde Pública da Universidade de São Paulo, São Paulo, SP, Brazil. Beneficiary of a fellowship for research productivity from the National Council for Scientific and Technological Development - CNPq, process/contract identification: 312656/2019-0, Brazil; l Institute of Infectious Diseases Emilio Ribas, São Paulo, Brazil; m Evandro Chagas Institute, Health of Ministry of Brazil, Belém, Pará, Brazil; Faculdade de Medicina da Universidade Federal do Pará, Brazil; n Unidad Procedimientos, Policlínico Neurología, Centro de Referencia de Salud Dr. Salvador Allende Gossens, Santiago de Chile, Chile; o Department of Pediatric Infectious Diseases and Immunology, School of Medicine, Pontificia Universidad Católica de Chile, Santiago de Chile, Chile; p Instituto de Salud Pública, Facultad de Medicina, Universidad Austral de Chile, Valdivia, Chile; q Hospital de San Fernando, Ministerio de Salud, San Fernando, VI Region, Chile; r Department of Pediatrics, Universidad Nacional de Colombia, Bogotá, DC, Colombia. Division of Infectious Diseases, HOMI, Hospital Pediátrico La Misericordia, Bogotá, DC, Colombia; s Hemera Unidad de Infectología IPS SAS, Bogota, Colombia; t Hospital San Vicente Fundación, Rionegro, Antioquia, Colombia; u Clinica Imbanaco Grupo Quironsalud, Cali, Colombia. Universidad Santiago de Cali, Cali, Colombia. Clinica de Occidente, Cali, Colombia. Clinica Sebastián de Belalcazar, Valle del Cauca, Colombia; v Epidemiology Unit, National Institute of Gastroenterology, La Habana, Cuba; w Hospital Salvador Bienvenido Gautier, Santo Domingo, Dominican Republic; x Pontificia Universidad Catolica Madre y Maestra (PUCMM), Santiago, Dominican Republic; y School of Medicine of the International University of Ecuador, Quito, Ecuador; z Universidad Técnica de Ambato, Ambato, Ecuador; aa Hospital Roosevelt, Guatemala City, Guatemala; ab Unit of Scientific Research, School of Medical, Faculty of Medical Sciences, Universidad Nacional Autónoma de Honduras (UNAH), Tegucigalpa, Honduras; ac Hospital Infantil de Mexico, Federico Gomez, Mexico City, Mexico; ad Departamento de Infectología, Hospital General de Tijuana, Tijuana, Mexico; ae Asociación de Microbiólogos y Químicos Clínicos de Nicaragua, Managua, Nicaragua; af Medicine Department-Infectious Diseases Service, Hospital Santo Tomas, Panama City, Panama. Instituto Oncologico Nacional, Panama city, Panama; ag University of Arizona College of Medicine-Phoenix, Division of Endocrinology, Department of Medicine.650 E Indian School Rd, suite 117B, Phoenix, AZ 85022, USA; ah Dirección de Investigación, Dirección Nacional de Vigilancia Sanitaria, Asunción, Paraguay; ai Division of Dermatology, Faculty of Medical Sciences, Universidad Nacional de Asunción, Asuncion, Paraguay; aj Infectious Diseases Division, Instituto Nacional de Salud del Niño San Borja, Lima, Peru. Facultad de Ciencias de la Salud, Universidad Privada de Tacna, Tacna, Peru; ak Universidad San Juan Bautista, Lima, Peru; al Unidad de Investigación para la Generación y Síntesis de Evidencias en Salud, Vicerrectorado de Investigación, Universidad San Ignacio de Loyola, Lima, Peru; am Hospital Evangélico de Montevideo, Montevideo, Uruguay; an Molecular Microbiology Laboratory, Department of Pathology, Molecular and Cell-based Medicine, Icahn School of Medicine at Mount Sinai, New York, USA; ao Centro de Investigaciones en Microbiología y Biotecnología-UR (CIMBIUR), Facultad de Ciencias Naturales, Universidad del Rosario, Bogotá, Colombia; ap Infectious Diseases Research Branch, Venezuelan Science Incubator and the Zoonosis and Emerging Pathogens Regional Collaborative Network, Cabudare, 3023, Lara, Venezuela; aq Faculty of Medicine, Universidad Central de Venezuela, Caracas, Venezuela; ar Biomedical Research and Therapeutic Vaccines Institute, Ciudad Bolivar, Venezuela; as Tropical Medicine Institute, Infectious Diseases Section, Universidad Central de Venezuela, Caracas 1053, Venezuela; at Investigador SNI Senacyt Panamá, Clinical Research Deparment, Instituto Conmemorativo Gorgas de Estudios de la Salud, Panama City, Panama

Still without ceasing the Coronavirus Disease 2019 (COVID-19) pandemic, a new viral threat has now emerged outside its endemic niche in Africa affecting multiple countries and continents. After its appearance in May 2022, a multi-country outbreak of monkeypox disease (MPX) has triggered significant concerns due to its rapid spread and potential for sexual transmission (as suggested by the detection of viral DNA in sexual fluids); this, in addition to the previously known transmission routes described throughout endemic countries of Africa over the last decades or the imported or travel-related cases reported since 2003.^1^

By June 28, 2022, at least 48 cases in seven Latin American countries (Argentina, Brazil, Chile, Colombia, Mexico, Peru, and Venezuela) have been PCR-confirmed, with at least 16 additional suspected cases (Figure 1).^2^ Most countries in the region have settled their epidemiological surveillance to detect probable and suspected cases according to national and international case definitions (by the World Health Organization). In addition, the Pan-American Health Organization has issued an epidemiological alert (https://bit.ly/3MUwYNI), with a series of considerations addressing the identification of cases, isolation, follow-up, contact tracing, clinical management, prevention and control. Nevertheless, multiple concerns have been raised, mainly from the healthcare sector, regarding currently available treatments and vaccination. Despite the absence of specific therapeutic alternatives for MPX, drugs with proven experimental efficacy and potential clinical impact such as cidofovir (especially its lipid conjugate brincidofovir) and tecovirimat, are not widely available in the region. Also, although MPX vaccination has been implemented for contacts of positive cases, at this stage, neither non-replicating/replicating-deficient live vaccinia virus-based vaccines with low reactogenicity, such as JYNNEOS®, nor classical anti-smallpox vaccines are available in most Latin American countries.

**Figure 1:**
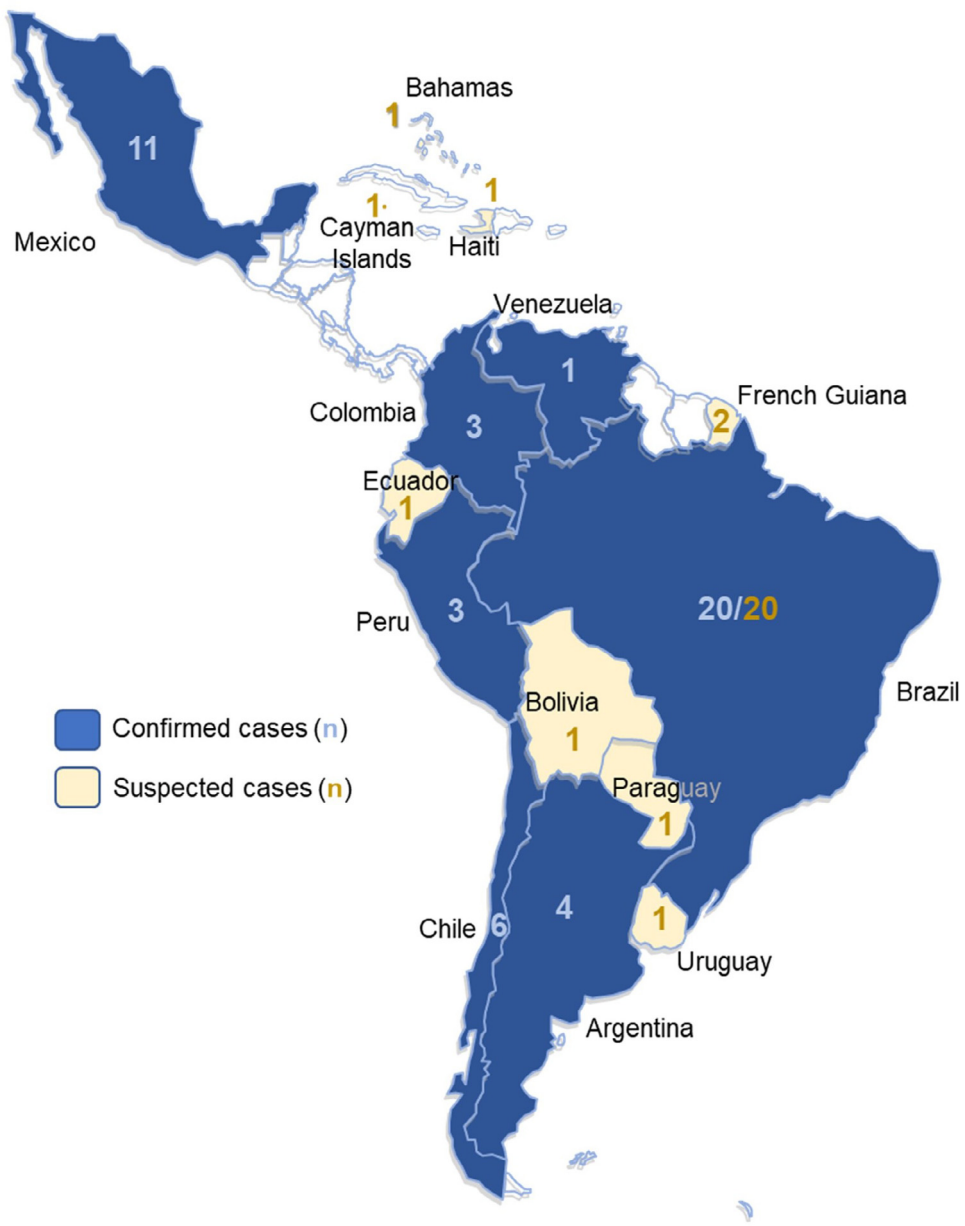
Current situation of MPX in Latin America (confirmed and suspected cases), up to June 28, 2022. Data sources: https://www.ilpandacentrostudio.it/uk.html, https://www.cdc.gov/poxvirus/monkeypox/response/2022/world-map.html.

On a more positive note, as a consequence of the COVID-19 pandemic, several countries have ramped up their current molecular testing capacity and have established broad laboratory networks sharing genomic surveillance data, resulting in better preparedness against other emerging threats such as the current MPX multi-country outbreak. Therefore, in addition to those countries throughout the region which have already confirmed cases, many others are also assessing suspected and probable cases through nucleic acid amplification tests (NAATs). Improvement in data integration between different sectors in the society, including healthcare and public health authorities, enhanced sanitary infrastructure, use of drugs with proven efficacy and safety, as well as the issuing of evidence-based guidelines in multiple countries has prevented a larger-scale expansion of the disease. Although it is expected that most cases of MPX will present as a mild disease, the last two and half years of the COVID-19 pandemic have positively impacted the quality of primary care interventions, build-up of intensive care units (ICU) capacities, equipment provisions and personnel training, among other advances.^3^ One of the great challenges and lessons learned from the pandemic relates to failures in risk communication. In this sense, strengthening epidemiological surveillance systems, and disseminating adequate information through reliable channels (official social media and web pages) with clear and assertive messages could contribute to gaining greater confidence from the broad public and assisting in the early case detection, thus halting transmission chains and preventing further outbreaks. Public health professionals, physician communities and organizations, healthcare authorities and scientific experts should combat misinformation and disinformation proactively based on clear, direct, culturally responsive messaging that is free of unnecessary scientific jargon.^4^

Despite the significant advances achieved as a consequence of the pandemic, the Latin American region still faces a complex scenario with multiple unfolding syndemics’, including communicable diseases, such as HIV infection, malaria, tuberculosis, orthohantavirus, arboviral diseases (particularly dengue, Zika, chikungunya, and yellow fever),^5^ among other endemic diseases. The still-prevalent large pockets of poverty present in the region are part of an unavoidable context that influences disease emergence having a higher impact in Latin America when compared to other high-income countries. More recently, this region has also witnessed the re-emergence of some vaccine-preventable diseases, such as chickenpox, a top differential diagnosis when assessing a suspected case of MPX. Thus, vaccination programs need to be enhanced in order to recover optimal coverage. In the context of the current preparedness, there is an urgent need for healthcare workers' education on the many clinical and epidemiological aspects of MPX, including considerations about its characterization and inclusion as a part of the differential diagnoses with other endemic diseases that may overlap with similar clinical findings,^6^ and even result in co-infections including other sexually transmitted pathogens^7^ like *Treponema pallidum* as well as other causative agents of endemic trepanomatosis like yaws (*T. pallidum subsp. pertenue*) and pinta (*T. carateum*) also known to be prevalent in Latin America.^8^

A major global concern among research and public health personnel relates to the risk and implications of the potential zoonotic transmission of MPX outside its endemic niche in several African countries. Can we have endemicity and animal hosts that may sustain further transmission cycles outside Africa? Some likely hosts, such as *Didelphis marsupialis, Monodelphis domestica* (opossums), *Sus scrofa domestica* (domestic pig), among others, have proved susceptible and are present in Latin America. These aspects of potential spillover to other susceptible species in the Americas deserve careful consideration and close monitoring.^9^ Several clinical, virological, and immunological aspects also deserve urgent research, mainly because, although the disease was discovered in humans in 1970, there is a significant knowledge gap as evidenced by recent bibliometric studies.

Despite its still moderate risk for expansion, allocation of necessary resources, strengthening epidemiological surveillance systems, and increased capacity building should be promptly prioritized in Latin American countries to detect imported cases and limit onward transmission (including autochthonous cases), which is expected to occur after the arrival imported cases. Latin America, fortunately, is now more prepared to confront new epidemic threats, as in the case of MPX.^10^

## Contributors

A.J. Rodriguez-Morales wrote the original draft. All authors contributed to reviewing and editing.

## Editor note

*The Lancet* Group takes a neutral position with respect to territorial claims in published maps and institutional affiliations.

## Declaration of interests

All authors declare no competing of interest. GC-M has received an independent grant from Pfizer through the Asociación Colombiana de Infectologia ACIN- capitulo central; speaker fees from Pfizer, Sanofi Pasteur and Biomerieux; and participated in Advisory Boards for MSD and Sanofi Pasteur.
